# Can glutathione be a biomarker for suicide risk in women 18 months postpartum?

**DOI:** 10.3389/fpsyt.2023.1142608

**Published:** 2023-02-09

**Authors:** Paula Michele da Silva Schmidt, Jéssica Puchalski Trettim, Aline Longoni, Mateus Grings, Mariana Bonati de Matos, Luciana de Avila Quevedo, Ana Paula Ardais, Fernanda Nedel, Gabriele Ghisleni, Guilhian Leipnitz, Ricardo Tavares Pinheiro, Adriano Martimbianco de Assis

**Affiliations:** ^1^Graduate Program in Health and Behavior, Center of Health Science, Universidade Católica de Pelotas - UCPel, Pelotas, Brazil; ^2^Graduate Program in Biological Sciences: Biochemistry, ICBS, Universidade Federal do Rio Grande do Sul - UFRGS, Porto Alegre, Brazil; ^3^Department of Biochemistry, Universidade Federal do Rio Grande do Sul - UFRGS, Porto Alegre, Brazil

**Keywords:** glutathione, antioxidants, psychiatric disorders, suicide risk, mood disorders, oxidative stress

## Abstract

**Background:**

Suicide risk is prominent among the problems affecting populations, mainly due to the broad family, psychosocial and economic impact. Most individuals at suicidal risk have some mental disorder. There is considerable evidence that psychiatric disorders are accompanied by the activation of neuro-immune and neuro-oxidative pathways. The aim of the study is to evaluate the serum levels of oxidative stress biomarkers in women at risk of suicide after 18 months of postpartum.

**Methods:**

This is a case-control study, nested within a cohort study. From this cohort, 45 women [15 without mood disorders and 30 with mood disorders (Major depression and Bipolar disorder)] were selected at 18 months postpartum, the depression and suicide risk were assessed using the Mini-International Neuropsychiatric Interview Plus (MINI-Plus) instrument, module A and C, respectively. Blood was collected and stored for later analysis of the reactive species (DCFH), superoxide dismutase (SOD), and glutathione reduced (GSH). For data analysis, the SPSS program was used. To compare the nominal covariates with the outcome GSH levels, the Student’s *t*-test or analysis of variance (ANOVA) was used. Spearman’s correlation was performed for analysis between the quantitative covariates and the outcome. To analyze the interaction between the factors, multiple linear regression was performed. Bonferroni analysis was used as an additional/secondary result to visualize differences in glutathione levels according to risk severity. After the adjusted analysis, *p*-values < 0.05 were considered statistically significant.

**Results:**

The percentage of suicide risk observed in our sample of women at 18 months postpartum was 24.4% (*n* = 11). After adjusting for the independent variables, only the presence of suicide risk remained associated with the outcome (β = 0.173; *p* = 0.007), low levels of GSH at 18 months after postpartum. Likewise, we verified the difference in GSH levels according to the degree of suicide risk, observing a significant association between the differences in glutathione means in the group of women with moderate to high risk compared to the reference group (no suicide risk) (*p* = 0.009).

**Conclusion:**

Our findings suggest that GSH may be a potential biomarker or etiologic factor in women at moderate to high risk of suicide.

## Introduction

Suicide takes a featured place among the problems that affect populations, mainly due to the broad family, psychosocial and economic impact. More than 800,000 people worldwide commit suicide annually and it is estimated that, for each completed case, there are more than 20 attempts. In Brazil, the suicide mortality rate is about 5.5 deaths per 100,000 inhabitants, with about 10,000 suicide deaths annually (1). While not every person who attempts suicide has a mental illness, the vast majority suffer from depression (2). It is estimated that the lifetime risk of suicide in people with depression is 6 to 15% (3), with a man’s risk of suffering from the disease being 11%, while that of a woman can reach 18.6% (2).

Interventions have emerged to prevent suicide, but what makes prevention difficult is precisely not knowing the situations that influence suicidal ideation or behavior. In addition, several studies have sought more effective alternatives for the drug treatment of individuals with suicidal behavior. Glutaminergic dysregulation has already been identified as a potential pathological pathway in psychiatric disorders, including depression and schizophrenia (4, 5). As well, oxidative damage and redox dysregulation appear to play important roles in the pathogenesis of psychiatric disorders due to the brain’s vulnerability to oxidative stress (6).

The central nervous system (CNS) is particularly sensitive due to the high rate of oxygen consumption, the high levels of polyunsaturated lipids (capable of undergoing lipid peroxidation) (7) and the auto-oxidation of some neurotransmitters, which can lead to the formation of reactive oxygen species (ROS) (8). In addition, the brain is quite vulnerable to oxidative damage, given its relatively low content of antioxidant defenses and the high content of metals (iron, zinc, magnesium and copper), which can catalyze the formation of reactive oxygen and nitrogen species (8).

Glutathione (GSH, L-γ-glutamyl-L-cysteinyl-glycine) is an endogenous antioxidant found in many tissues, however, in the brain, it plays a major role and is widely used by neurons to neutralize oxidative stress and maintain neural cell functionality and viability adequate (9). However, low concentrations of GSH have been reported in some of the major psychiatric disorders (10, 11), including major depressive disorder (MDD) (12), bipolar disorder (13), and schizophrenia (14–18).

Furthermore, the literature shows that stressful conditions (shock) increase brain energy demand, which results in an increase in ROS and consequently a decrease in GSH levels in the cerebral cortex of mice, an effect that was reversed by antidepressants (19). GSH may be an endogenous neuromodulator of mood (20, 21). Thus, our study hypothesis is that women with suicidal risk present cerebral redox imbalance resulting in a decrease in blood GSH levels. Therefore, the objective was to evaluate the serum levels of oxidative stress biomarkers in women at risk of suicide after 18 months of postpartum.

## Materials and methods

### Design

This is a case-control study nested within a population-based cohort study, conducted within a city in the south of Brazil. The cohort project to which this case is linked was approved by the committee of research ethics of the Catholic University of Pelotas, under-report number 1.729.653. For more details on sample capturing, read the publications of Pinheiro et al. (22, 23).

**FIGURE 1:**
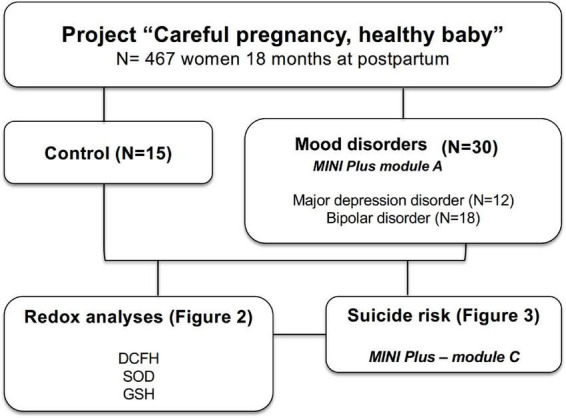
Study organization chart. Women (control and mood disorder) were selected from the cohort entitled “Careful pregnancy, healthy baby” for analysis of plasma biomarkers of oxidative stress and risk of suicide.

For this study, 45 women [15 without mood disorders (control) and 30 with mood disorders (case)] from the central project were selected, all of them participating in the 18-month postpartum phase and coming from an initial selection (case) that considered a diagnosis of mood disorders (Figure 1).

### Instruments

The instrument used to assess depression and the risk of suicide in this research was the Mini-International Neuropsychiatric Interview Plus (MINI-Plus 5.0.0 Brazilian version), module A and module C, respectively (24). The MINI-Plus is a brief standardized diagnostic interview (15–30 min), compatible with the criteria of the diagnostic and statistical manual of mental disorders (DSM-IV) and the international classification of diseases (ICD-10), which is intended for use in clinical practice and research in primary care and psychiatry. The suicidality section inquires about several components of suicide risk with the following questions: over the last month: (1) Have you wished you were dead? (score: 1 point); (2) Have you wanted to harm yourself? (2 points); (3) Have you thought of committing suicide? (6 points); (4) Have you planned how to commit suicide? (10 points); (5) Have you attempted suicide? (10 points), and (6) Have you ever attempted suicide? (4 points). The risk for suicide range was “low” (score 1–5), “moderate” (score 6–9), and “high” (score eN10). For analysis, the scores were dichotomized as absent (low or absent risk) or presence (moderate or high risk), as recommended by the MINI-Plus author (25, 26). The suicidality module of MINI-Plus is largely used with adequate validity and reliability (27, 28). Women variables, like age and schooling (collected in completed years and later categorized in terciles), were collected *via* questions of the structured general questionnaire.

### Blood sample collection and processing

The collection of biological material to assess the redox assays was performed by venipuncture and stored at −80°C for further analysis of blood parameters (29).

### Redox assays

#### Glutathione reduced

The GSH dosage was analyzed according to Browne and Armstrong (30). Samples (approximately 0.09 mg of protein) were treated with 2% metaphosphoric acid (1:1) and centrifuged at 7,000 *g* for 10 min. After deproteination, an aliquot of supernatant (30 μL) was then added to a medium containing 185 μL of 100 mM sodium phosphate buffer, pH 8.0, with 5 mM EDTA, and 15 μL o-phthaldialdehyde (1 mg/ml in methanol), and incubated for 15 min at room temperature in a dark room. The fluorescence was measured at 350 (excitation) and 420 (emission) nm. A calibration curve was prepared using a GSH standard solution (0.001–1 mM) and the results were expressed as nmol GSH/mg protein.

#### 2’,7’-dichlorofluorescein assay

To assess reactive species levels, 2′,7′-dichlorofluorescein (DCFH-DA) was used as a probe (29). Sixty microliters of the diluted sample were incubated at 37°C in the dark for 30 min, with the addition of 240 μL of DCFH diacetate (DCFH-DA) in a 96-well plate. DCFH-DA was cleaved by cellular esterases and formed DCFH, a non-fluorescent compound that was oxidized by reactive species present in the sample, producing a fluorescent compound, DCF. Several one-electron-oxidizing species will oxidize DCFH to DCF including hydroxyl radicals (^⋅^OH), compounds I and II formed from peroxidase or heme interaction with H_2_O_2_, ^⋅^NO_2_ formed from the myeloperoxidase/H_2_O_2_/NO_2_^–^ system, hypochlorous acid (HOCl), and reactive species formed from peroxynitrite (ONOO^–^/ONOOH) decomposition (29). DCFH oxidation was fluorometrically measured using a 488 nm excitation and 525 nm emission wavelength. A standard curve, using standard DCF (0.25–10 mM), was performed in parallel with the samples, and the results were expressed as nmol/mg protein.

### Superoxide dismutase activity

Superoxide dismutase (SOD) (EC 1.15.1.1) activity was assessed by quantifying the inhibition of the superoxide-dependent autoxidation of epinephrine and analyzing the absorbance of the samples at 480 nm. In microplate wells containing a sample (30 μL–60 μg of protein), 140 μL of glycine buffer (50 mM; pH 10.2) and 10 μL of catalase (EC 1.11.1.6) (10 μM) were added. In the standard wells, only 180 μL of glycine buffer (50 mM; pH 10.2) and 10 μL of catalase (10 μM) were added. The reaction was initiated by adding 10 μL of epinephrine (60 mM) in all wells. Zero time absorbance was taken at 480 nm, followed by recording the absorbance after 10 min at 32°C. SOD activity was defined as the amount of enzyme required to inhibit the oxidation of epinephrine by 50%. The data were calculated as units/mg protein (29).

### Statistical analysis

After coding the instruments, double data entry was performed in the EpiData 3.1 program to test typing inconsistencies. The statistical package for the social sciences program was used for data analysis. To compare the nominal covariates (economic level and suicide risk) with the outcome glutathione levels, the Student’s *t*-test or ANOVA was used. Spearman’s correlation was performed for analysis between the quantitative covariates (age and education) and the outcome. To analyze the interaction between the factors, multiple linear regression was performed. Bonferroni analysis was used as an additional/secondary result to visualize differences in glutathione levels according to risk severity. After the adjusted analysis, *p*-values < 0.05 were considered statistically significant.

## Results

The distribution of the sample is shown in Table 1. The prevalence of suicide risk in the mood disorder group is 36.7% (20% low risk and 16.7% moderate/severe risk). The suicide risk was associated with a mood disorder (*p* < 0.05). The raw analysis showed no significant difference in suicide risk according to age, skin color, and schooling (*p* > 0.05).

**TABLE 1:** Sample characteristics of women (control or mood disorder) at risk of suicide.

	Control *N* (%)	Mood disorder *N* (%)	*p*-value
Age (years)			1.000
Up to 23	3 (20.0)	6 (20.0)	
24–29	6 (40.0)	12 (40.0)	
30 or more	6 (40.0)	12 (40.0)	
Skin color*			0.304
White	10 (76.9)	15 (57.7)	
Non-white	3 (23.1)	11 (42.3)	
Schooling			0.236
8 years or less	14 (93.3)	23 (76.7)	
9 years or more	1 (6.7)	7 (23.3)	
Suicide risk score			0.026
Without	15 (100.0)	19 (63.3)	
Low	0 (0.0)	6 (20.0)	
Moderate/severe	0 (0.0)	5 (16.7)	
Total	15 (100.0)	30 (100.0)	

*Variable with missing.

**FIGURE 2:**
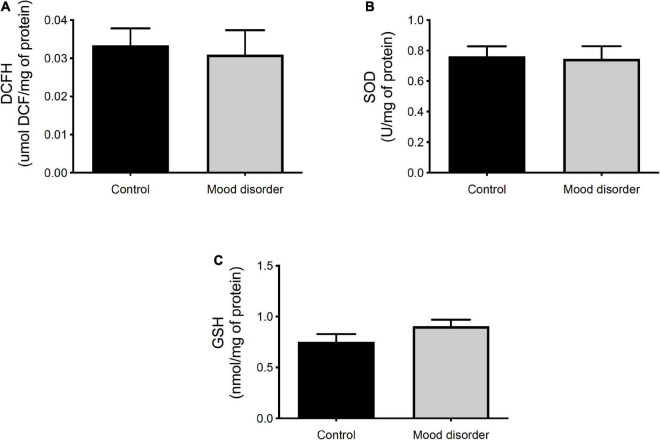
Oxidative stress analysis in plasma of women (control, *N* = 15 and mood disorder, *N* = 30) at 18 months postpartum. **(A)** DCFH is expressed as μmol/mg of protein; **(B)** superoxide dismutase (SOD) activity is expressed as U/mg of protein; and **(C)** GSH content is expressed as nmol/mg of protein. Statistical analysis was analyzed by *t*-test.

In Figure 2, we compare the redox status in the serum of women without (control) or with a mood disorder and did not observe any statistical difference in reactive species analyses (Figure 2A), SOD activity (Figure 2B), and glutathione reduced (Figure 2C).

Analyzing Table 1 of the total number of women participating in this study (*n* = 45), the mean age was 27.67 years (SD1 = 5.372; minimum = 17; maximum = 37), schooling had a mean of 11.58 complete years of schooling (SD = 3.876) and the predominant social class in our sample was C (71.1%; *n* = 32). The percentage of suicide risk observed in our sample of women at 18 months postpartum was 24.4% (*n* = 11) and of women without suicide risk was 75.6% (*n* = 34). Regarding the bivariate analysis, Table 2 also shows an association between GSH levels and the risk of suicide (*p* = 0.002).

**TABLE 2:** Analysis of the serum level of glutathione according to age, education in years, social class (A/B, C, and D/E), and risk of suicide diagnosed by MINI-Plus (Yes/No) of women at 18 months postpartum.

Variables		Glutathione reduced					
		Bivariate analysis			Multivariate analysis		
	Average (± SD)	*R*		*p*-value	β	CI 95%	*p*-value
Age (years)	27.67 (5.37)	0.29	–	0.049	0.007	−0.003; 0.017	0.165
Education (in years of study)	11.58 (3.88)	−0.67	–	0.660	−0.003	−0.020; 0.014	0.747
		***N* (%)**	**Average**	**SD**			
Social class				0.117	0.040	0.081; 0.161	0.510
A/B	08 (17.8)	−0.08	0.18	–	–	–	–
C	32 (71.1)	−0.14	0.19	–	–	–	–
D/E	05 (11.1)	0.04	0.08	–	–	–	–
Suicide risk				0.002*	0.173	0.050; 0.296	0.007*
No	34 (75.6)	−0.15	0.17	–	–	–	–
Yes	11 (24.4)	0.04	0.14	–	–	–	–

*Significance level 5%. SD, standard deviation; CI, confidence interval.

Again, in Table 2, all independent variables were taken into multiple analyses by linear regression to adjust for the effect of suicide risk (primary exposure) on glutathione levels (outcome). After adjustment, only the presence of risk of suicide remained associated with the outcome (β = 0.173; *p* = 0.007).

**FIGURE 3:**
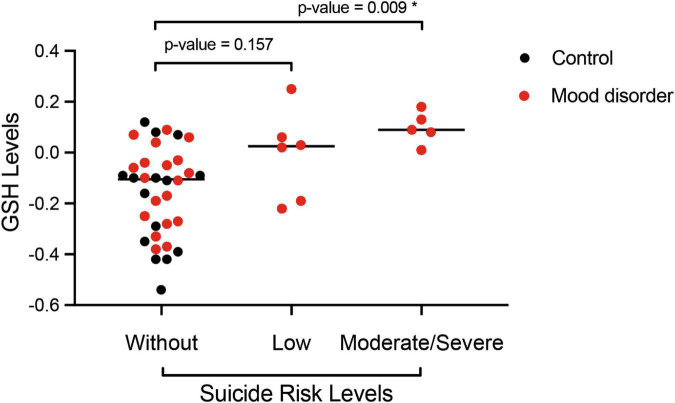
*Post hoc* analysis by Bonferroni of the serum glutathione reduced (GSH) level according to the degree of suicide risk classified by MINI-Plus module C (without, low, and moderate/severe risk) of women (control, *N* = 15 and mood disorder, *N* = 30) at 18 months postpartum. *p < 0.05 indicates a significant difference.

According to the degree of suicide risk, 13.3% (*n* = 6) of the women had a low risk of suicide and 11.1% (*n* = 5) had a moderate to high risk. In Figure 3, as an additional analysis by Bonferroni, we verified the differences in glutathione levels according to the degree of suicide risk, observing a significant association between the differences in glutathione means in the group of women with moderate to high risk compared to the reference group (without risk of suicide).

## Discussion

Unfortunately, the incidence of suicide is increasing and now represents the leading cause of mortality for people aged 15 to 44 years (3). Our sample was within this age group and this may explain the high prevalence of women at risk of suicide in this study. It is also worth mentioning that the selection of women for our study was carried out by convenience since the participants were part of another follow-up study, which affected the study’s sample size, which may be these limitations for the interpretation of our results. However, the present work presents promising results from the translational point of view, where we confirm the hypothesis of the study demonstrating that women with suicide risk (moderate to high) present cerebral redox imbalance resulting in a decrease in blood GSH levels.

While the risk of death and suicide attempts is lower during and shortly after pregnancy than in the general female population, suicides account for up to 20% of all postpartum deaths and represent a leading cause of mortality in the peripartum period, which corroborates the high prevalence of suicide risk in our sample of women (24). According to Hirst and Moutier (24), the transition to parenthood is a stressful event in life, and exposure to such events can trigger the first episodes of mania or potentially severe mixed states. Furthermore, the increased risk of suicide is related to stressors such as life events (25). The percentage of women in our sample at risk of suicide (24.4%) at 18 months postpartum was of concern from the point of view of women’s health.

Under basal physiological conditions, there is a controlled balance between pro-oxidant molecules and antioxidant molecules, and an imbalance between these molecules is called oxidative stress (31). Glutathione is the brain’s main antioxidant, and recent post-mortem and genetic data support its involvement in the pathophysiology of bipolar disorder (20, 32, 33). GSH is therefore a sensitive and reliable endogenous marker of oxidative stress. A post-mortem study showed lower levels of GSH in the prefrontal cortex of patients with MDD, bipolar disorder, and schizophrenia when compared to healthy controls (34). Our study found lower levels of GSH in women at risk of suicide. In line with our result, in 2017, Freed et al. (20) suggested that a lower GSH may be a potential marker of MDD early in the course of the disease. GSH levels in the occipital cortex were lower in adolescents with MDD compared to controls. It should also be noted that MDD in adolescents is associated with a high risk of suicide (32).

Other studies examining blood serum and plasma GSH concentrations have also identified significantly lower GSH in MDD patients compared with healthy patients (33, 35). In addition, lower brain levels of GSH were found in rodents with symptoms of depression (36, 37). More relevantly, using magnetic resonance imaging, Shungu et al. (38) measured and then compared occipital cortex (COC) GSH levels in unmedicated adults with MDD to healthy participants and found 21% lower GSH levels in MDD patients. Similarly, another recent 1H MRS study reported lower *in vivo* levels of GSH in the COC of unmedicated adults with MDD versus healthy controls (39). When we evaluated the degree of suicide risk, we found that serum GSH levels were significantly lower for moderate to high suicide risk than for women without suicide risk, suggesting an association of glutathione with the degree of suicide risk. However, in contrast to our findings, for Freed et al. (20), glutathione levels did not correlate with MDD severity.

Oxidative damage and redox dysregulation appear to play important roles in the pathogenesis of psychiatric disorders due to the brain’s vulnerability to the toxic effects of oxygen-free radicals (31). Regarding studies of potential treatments, N-acetylcysteine (NAC) is believed to exert therapeutic antioxidant effects as a substrate for glutathione synthesis. NAC readily crosses the blood-brain barrier providing a cysteine substrate for GSH synthesis in the brain, in addition to acting directly as a scavenger of ROS (40). Hans et al. (41) suggest that NAC may have potential use as an adjunct to fast-acting treatment in MDD. Although preliminary, our findings appear to imply reduced glutathione as a potential biomarker or etiologic factor among women at risk of suicide, with therapeutic implications. For example, NAC may be one such therapeutic strategy as it restores GSH and has been investigated as a therapeutic agent in adults with various neuropsychiatric disorders (40, 42). According to the results of a recent meta-analysis, NAC was moderately effective in relieving depression symptoms in adults with MDD, bipolar disorder, and other psychiatric conditions (43, 44). Furthermore, NAC directly evidenced antidepressant-like effects in rodent models of depression through its role as an antioxidant (45, 46).

Mechanisms of oxidative stress have been implicated in the pathogenesis of psychiatric disorders (31). This hypothesis has a theoretical appeal, as the brain is considered particularly vulnerable to oxidative damage for several reasons. These include its comparatively high utilization of oxygen and therefore generation of free radical byproducts, its modest antioxidant defenses, its lipid-rich constitution that provides substrates ready for oxidation, the reducing potential of certain neurotransmitters, and the presence of redox-catalytic metals, like iron and copper (47). This intrinsic oxidative vulnerability of the brain and growing evidence of neurodegenerative changes associated with many psychiatric syndromes suggests that oxidative damage may be a plausible pathogenic candidate. We can see that women with reduced GSH levels who demonstrate a risk of moderate/severe suicide were in the mood disorder group, despite knowing that psychiatric disorders alter the brain redox state (31), the effect observed in our study (GSH levels) is not only of these disorders but related to the risk of suicide (mainly outcome), since most women in the mood disorder group are at no risk for suicide and without changes in GSH levels.

## Conclusion

Our work had some limitations, including the small sample size, the selection by the convenience of sample for the study, and the lack of follow-up of women’s GSH levels at other time points (longitudinal study). However, the present study has contributed evidence in support of the role of oxidative stress in mood disorders and is the first study to our knowledge that examined GSH in women at risk for suicide. If replicated in a larger sample, the present finding of GSH deficit in women at risk of suicide may provide important information for the development of new paradigms of assessment, prevention, and treatment. As stated earlier, although preliminary, these findings seem to imply GSH as a potential biomarker or etiologic factor among women at moderate to high risk of suicide, and according to the literature, with therapeutic implications. For example, NAC, which restores GSH, has been investigated as a therapeutic agent in adults with various neuropsychiatric disorders. Since the mechanisms associated with the risk of suicide may differ in the episodes presented. Future studies evaluating glutathione with a larger sample size with statistical power to differentiate the association proposal by manic or depressive episodes, as well as the use of NAC as a suicide risk reducer, seem justified.

## Data availability statement

The original contributions presented in this study are included in this article/supplementary material, further inquiries can be directed to the corresponding author.

## Ethics statement

The studies involving human participants were reviewed and approved by the Comitê de Ética em Pesquisa da Universidade Católica de Pelotas. Written informed consent to participate in this study was provided by the participants or their legal guardian/next of kin.

## Author contributions

All authors listed have made a substantial, direct, and intellectual contribution to the work, and approved it for publication.
